# Clinical utility of paced finger tapping assessment in idiopathic normal pressure hydrocephalus

**DOI:** 10.3389/fnhum.2023.1109670

**Published:** 2023-02-23

**Authors:** Yoko Shimizu, Motoki Tanikawa, Mitsuya Horiba, Kento Sahashi, Shoji Kawashima, Akihiko Kandori, Tomoyasu Yamanaka, Yusuke Nishikawa, Noriyuki Matsukawa, Yoshino Ueki, Mitsuhito Mase

**Affiliations:** ^1^Department of Rehabilitation Medicine, Nagoya City University Graduate School of Medical Sciences, Nagoya, Japan; ^2^Department of Neurosurgery, Nagoya City University Graduate School of Medical Sciences, Nagoya, Japan; ^3^Department of Neurology and Neuroscience, Nagoya City University Graduate School of Medical Sciences, Nagoya, Japan; ^4^Hitachi, Ltd., Research and Development Group, Center for Exploratory Research, Tokyo, Japan

**Keywords:** idiopathic normal pressure hydrocephalus, finger tapping, diagnosis, 2.0 Hz, cut-off value

## Abstract

**Background:**

The Finger Tapping (F-T) test is useful for assessing motor function of the upper limbs in patients with idiopathic normal pressure hydrocephalus (iNPH). However, quantitative evaluation of F-T for iNPH has not yet been established. The purpose of this study was to investigate the usefulness of the quantitative F-T test and optimal measurement conditions as a motor evaluation and screening test for iNPH.

**Methods:**

Sixteen age-matched healthy controls (mean age 73 ± 5 years; 7/16 male) and fifteen participants with a diagnosis of definitive iNPH (mean age 76 ± 5 years; 8/15 male) completed the study (mean ± standard deviation). F-T performance of the index finger and thumb was quantified using a magnetic sensing device. The performance of repetitive F-T by participants was recorded in both not timing-regulated and timing-regulated conditions. The mean value of the maximum amplitude of F-T was defined as M-Amplitude, and the mean value of the maximum velocity of closure of F-T was defined as cl-Velocity.

**Results:**

Finger Tapping in the iNPH group, with or without timing control, showed a decrease in M-Amplitude and cl-Velocity compared to the control group. We found the only paced F-T with 2.0 Hz auditory stimuli was found to improve both M-Amplitude and cl-Velocity after shunt surgery.

**Conclusion:**

The quantitative assessment of F-T with auditory stimuli at the rate of 2.0 Hz may be a useful and potentially supplemental screening method for motor assessment in patients with iNPH.

## Highlights

- Patients with idiopathic normal pressure hydrocephalus (iNPH) have not only the lower limb dysfunction but also the upper limb.
- This is the first report of quantitative evaluation of finger tapping (F-T) movements in patients with iNPH under multiple conditions.
- The results showed particularly that patients had impaired hand dexterity compared to controls, and cut-off values were calculated.
- Quantitative assessment of F-T with 2.0 Hz auditory stimulation may be a useful and complementary screening method for motor assessment of patients with iNPH.

## Introduction

Normal pressure hydrocephalus (NPH) was first described in 1965 by Hakim and Adams as a syndrome accompanied by a triad of progressive symptoms: gait disturbance, cognitive impairments and urinary incontinence (Adams et al., 1965). The assessment of gait is fundamental to diagnose idiopathic NPH (iNPH), because gait disturbance is typically the first clinical symptom (Ojemann et al., 1969). The assessment of gait after drainage is very important for predicting functional improvement after shunt surgery (Williams et al., 2008), but it is sometimes difficult if patients have heavily deteriorated motor function. However, gait assessment in iNPH is sometimes difficult because the gait is affected by various factors such as orthopedic diseases, motor weakness and psychiatric apathy (Kito et al., 2009; Allali et al., 2017). The CSF tap test, predictive of post-shunt improvement, involves repeated assessments of motor and cognitive function. Therefore, the ideal supplemental functional evaluation of the tap test should be as reproducible as possible and cause as little physical and mental to put burden on the patient as possible, such as falls.

The pattern of gait disturbance in NPH is often described as lower-body parkinsonism according to the guidelines for diagnosis (Espay et al., 2008). Kang et al. (2016) reported that parkinsonism of iNPH is present not only in the lower limbs but also the upper limbs. Previous studies reported that upper limb dysfunction in patients with iNPH was similar to that of bradykinesia symptoms in Parkinson’s disease (PD) (Krauss et al., 1997; Nowak et al., 2006). Repetitive index finger on thumb tapping is considered an appropriate evaluation of bradykinesia because upper limb impairments in PD are reported to be more apparent distally than proximally (Agostino et al., 1998; Nowak and Topka, 2006). Stegemöller et al. (2017) explained that repetitive movements in Parkinsonism are characterized by reductions in amplitude (hypokinesia), velocity (bradykinesia), and rhythmicity, with or without hesitations and arrests. Several previous studies have reported that patients with iNPH had upper limb dysfunction (Sørensen et al., 1986; Nowak and Topka, 2006; Tsakanikas et al., 2009; Hellström et al., 2012), and some found finger tapping (F-T) to be particularly useful in assessing motor function of iNPH (Lenfeldt et al., 2008; Liouta et al., 2017; Behrens et al., 2019). The assessment of motor function of the upper limbs might be helpful in diagnosing NPH to avoid falls and difficulties in gait assessment.

Although the usefulness of F-T test has widely been accepted, its quantitative evaluation for iNPH has not yet been established. Notably, previous studies reported that impairments in simple repetitive finger movements was observed at rates near and above 2.0 Hz in patients with PD (Pastor et al., 1992; Stegemöller et al., 2009). Based on these reports, the present study used 1.0 and 2.0 Hz auditory stimuli in the timing-regulated condition and spontaneous motor tempo (SMT) and maximum effort (as quickly and as big as possible) in the not timing-regulated condition (Christopher et al., 2008; Dawn et al., 2020).

This is the first report evaluating repetitive F-T of iNPH patients quantitatively with multiple conditions. The purpose of this study was to find the optimal conditions for quantitative evaluation of repetitive F-T to help assess the function of iNPH patients.

## Materials and methods

### Subjects

Sixteen age-matched healthy controls (mean age 73 ± 5 years; 7/16 male) and 15 participants with a diagnosis of definitive iNPH (mean age 76 ± 5 years; 8/15 male) completed the study (mean ± standard deviation). After shunt surgery, the clinical symptoms of patients with iNPH improved significantly and they received a definitive diagnosis of iNPH. All participants were right-handed and completed the Edinburgh Handedness Inventory and Mini-Mental State Examination Japanese (MMSE-J). We excluded participants with conditions such as severe hearing loss or other diseases that could interfere significantly with the test.

We used the iNPH Grading Scale (iNPHGS) to evaluate the triad of disease severity (Kubo et al., 2008). The iNPHGS consists of three domains: gait disturbance, cognitive impairment and urinary incontinence, each of which is scored according to five levels (0 to 4, respectively).

The walking ability of all patients was also measured for the severity of symptoms by the Timed Up and Go Test (TUG), which is accepted as a valid test to assess motor function (Podsiadlo and Richardson, 1991). The study protocol was approved as prospective study by the Nagoya City University Institutional Review Board, which is compliant with the guidelines in the Declaration of Helsinki. Informed consent was obtained for all participants (patients and controls) (ID: 60-18-0213).

### Parameters of F-T

**FIGURE 1:**
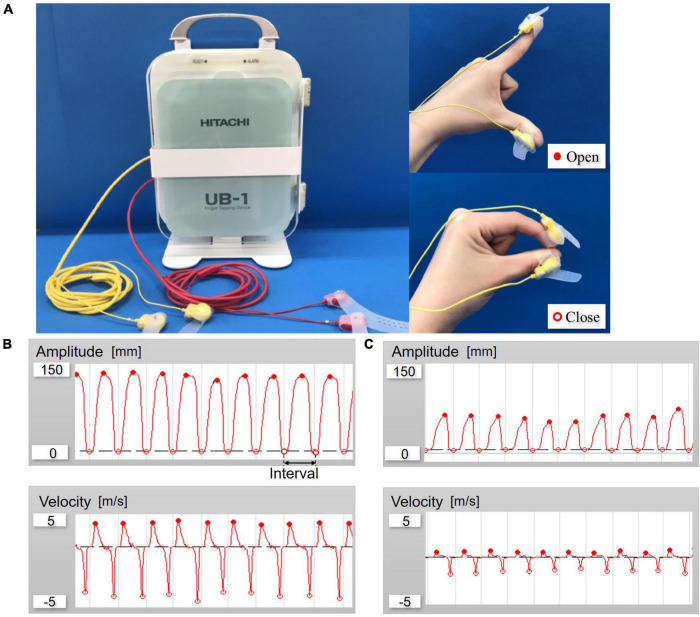
**(A)** Overall view of UB-1; Finger-tapping system with magnetic sensors and finger tap. **(B)** Example of waveforms control. **(C)** Example of waveforms patient with idiopathic normal pressure hydrocephalus (iNPH) (amplitude and velocity).

Finger-tapping performance of the paced index finger and thumb was quantified using a magnetic sensing device (UB-1, HITACHI Ltd., Tokyo, Japan) (Figure 1). Participants tapped their index finger on the thumb while wearing magnetic sensors. The device can automatically detect and record the amplitude, velocity and interval of each a finger tap. Since a previous study described repetitive movements in parkinsonism as being characterized by reductions in amplitude, velocity and rhythmicity (Stegemöller et al., 2017), we chose these parameters for evaluation of F-T in patients with iNPH. The mean of the maximum amplitude was defined as M-Amplitude, and the mean of the maximum velocity of the closed motion was defined as cl-Velocity. The variability was calculated as the coefficient of variation (CV) of each parameter, which were M-Amplitude, cl-Velocity, and tapping interval.

### Recording of F-T

First, the maximum distance from index finger to thumb at rest was measured by each participant and defined as r-Amplitude as one of the calibrations before the F-T evaluation. We then recorded the performance of repetitive F-T both not timing-regulated and timing-regulated conditions by participants for 15 s each of the non-dominant and the dominant hand. F-T test was performed in the order of at a spontaneous pace, at rates of 1.0 and 2.0 Hz by auditory stimulation with a metronome, and at the maximum effort pace (as quickly and as big as possible). Participants were instructed to close their fingers together when auditory stimuli were given. Furthermore, the patients were evaluated for F-T before and after shunt surgery.

### Statistics

We computed the sample size according to the of F-T test we considered most relevant in our investigation, finger oscillation test during which recorded the speed of tapping (Reitan and Wolfson, 1993). We calculated the effect size from the mean and standard deviation of the F-T speeds of controls and iNPH according to Lenfeldt et al. (2008), and be aimed to detect a change of more than 30% of it. To detect this decline with a two-tailed type I error of 0.05 and a power of 80%, the estimated sample size was 20 persons (10 persons in each group). In order to account for the possibility of some patients not completing the study, we aimed to enroll 15 subjects in each group. We used a two-way repeated measures analysis of variance (ANOVA) to compare on all parameters of the F-T test, with non-dominant and dominant hand as the within factor and disease as the between factor. We used a paired *t*-test for M-Amplitude and cl-Velocity, and Wilcoxon signed-rank test for CV to compare the parameters of the F-T test in patients before and after surgery with the mean values of the dominant and non-dominant hand, except for CV of cl-Velocity. Since the normality of M-Amplitude and cl-Velocity was confirmed by the Shapiro-Wilk test, the cut-off thresholds for the parameter of paced F-T were calculated from ROC analyses, using the area under the curve (AUC) and determined to a 95% confidence interval (CI). The Pearson correlation coefficient was calculated for continuous variables between the parameters of F-T test and other function in patients with iNPH. The results were expressed as mean ± standard deviation of the mean. Statistical analysis was completed with Statistical software JMP^®^10.0.2 (Statistical Analysis Software, SAS Institute Inc., Charlotte, NC, USA). *p*-values less than 0.05 were considered as statistically significant.

## Results

### Clinical characteristics

Characteristics of participants are provided in Table 1. There were no significant differences in age and gender between groups. However, MMSE-J scores were significantly lower in patients with iNPH as compared to healthy controls (*P* < 0.001). The mean iNPHGS of gait impairment scores improved from 2.00 ± 0.65 to 1.20 ± 1.10 (*P* < 0.01) and the mean iNPHGS of urination incontinence scores from1.46 ± 0.83 to 1.10 ± 0.90 (*P* < 0.05) after shunt surgery, respectively. The mean iNPHGS for cognitive impairment scores showed a tendency for improvement, from1.93 ± 0.80 to 1.80 ± 1.00 (*P* = 0.08).

**TABLE 1:** Characteristics of participants.

			Date are mean ± SD
	**Control**	**iNPH**	***P*-value**
Age	73.1 ± 5.1	76.7 ± 5.9	N. S
Sex (male/female)	7/9	8/7	N. S
MMSE-J	28.6 ± 1.6	23.6 ± 3.5	*p* < 0.001
Evans index	–	0.35 ± 0.03	–
Duration (years)	–	2.26 ± 1.6	–

MMSE-J, Mini Mental State Examination-Japanese; NS, not significant; SD, standard deviation.

### F-T performance

The comparison of paced F-T performance between controls and patients is shown in Table 2.

**TABLE 2:** Comparison of F-T parameters in controls and patients with definite iNPH.

	Groups				ANOVA results					
	**Controls**		**iNPH**		**Difference between groups**		**Non-/dominant hand**		**Group × dominant hand**	
	**Non-dominant hand**	**Dominant hand**	**Non-dominant hand**	**Dominant hand**	***F***	***P*-value**	***F***	***P*-value**	***F***	***P*-value**
**Frequency of taps (Hz)**										
SMT	2.29 ± 0.45	2.35 ± 0.51	1.58 ± 0.62	1.57 ± 0.60	25.37	<0.001⁂	0.32	0.58	0.02	0.90
Maximum effort	3.19 ± 0.59	3.47 ± 0.66	2.84 ± 0.72	2.76 ± 0.80	6.48	0.01	1.24	0.27	0.27	0.61
1.0 Hz	1.00 ± 0.01	1.00 ± 0.01	1.00 ± 0.05	0.95 ± 0.17	0.44	0.51	0.60	0.44	0.52	0.48
2.0 Hz	2.00 ± 0.01	2.00 ± 0.01	1.93 ± 0.27	1.97 ± 0.09	1.62	0.28	0.23	0.63	0.20	0.65
**M-Amplitude of finger tapping (mm)**										
SMT	91.29 ± 22.83	98.59 ± 21.20	91.23 ± 38.68	83.05 ± 35.73	1.02	0.31	<0.001	0.95	1.01	0.32
Maximum effort	103.31 ± 27.81	98.46 ± 23.00	70.13 ± 29.89	68.10 ± 24.74	22.31	<0.001⁂	0.26	0.61	0.04	0.83
1.0 Hz	102.26 ± 13.06	101.29 ± 20.66	84.57 ± 36.84	81.78 ± 36.55	6.70	0.01*	0.07	0.80	0.02	0.90
2.0 Hz	80.21 ± 17.44	84.15 ± 14.07	51.84 ± 28.11	54.15 ± 27.48	8.85	<0.001⁂	0.12	0.73	0.15	0.70
**CV (variability) of M-Amplitude**										
SMT	0.10 ± 0.07	0.07 ± 0.03	0.14 ± 0.13	0.13 ± 0.08	4.85	0.03*	1.09	0.30	0.36	0.55
Maximum effort	0.13 ± 0.10	0.09 ± 0.05	0.22 ± 0.10	0.18 ± 0.09	15.82	<0.001⁂	3.45	0.07	<0.001	0.97
1.0 Hz	0.07 ± 0.03	0.06 ± 0.03	0.13 ± 0.08	0.10 ± 0.05	16.01	<0.001⁂	2.12	0.15	0.58	0.44
2.0 Hz	0.11 ± 0.05	0.09 ± 0.03	0.23 ± 0.09	0.18 ± 0.07	18.39	<0.001⁂	1.61	0.20	0.06	0.81
**cl-Velocity of finger tapping (M/s)**										
SMT	1.72 ± 0.39	1.85 ± 0.39	1.14 ±0.40	1.22 ± 0.39	37.12	<0.001⁂	1.04	0.31	0.07	0.79
Maximum effort	1.87 ± 0.41	2.01 ± 0.43	1.29 ± 0.47	1.37 ± 0.37	31.74	<0.001⁂	1.05	0.31	0.07	0.79
1.0 Hz	1.63 ± 0.38	1.71 ± 0.42	1.15 ± 0.50	1.20 ± 0.55	17.23	<0.001⁂	0.32	0.57	0.02	0.89
2.0 Hz	1.66 ± 0.39	1.78 ± 0.37	0.99 ± 0.51	1.07 ± 0.44	12.91	<0.001⁂	0.99	0.33	0.04	0.85
**CV (variability) of cl-Velocity**										
SMT	0.12 ± 0.04	0.09 ± 0.03	0.23 ± 0.06	0.17 ± 0.07	49.45	<0.001⁂	9.18	<0.001⁂	0.95	0.34
Maximum effort	0.19 ± 0.15	0.12 ± 0.04	0.26 ± 0.09	0.19 ± 0.08	7.88	0.01*	6.50	0.01*	<0.001	0.99
1.0 Hz	0.10 ± 0.05	0.11 ± 0.05	0.24 ± 0.11	0.20 ± 0.10	32.26	<0.001⁂	0.99	0.32	1.09	0.30
2.0 Hz	0.12 ± 0.06	0.09 ± 0.04	0.28 ± 0.12	0.21 ± 0.11	17.56	<0.001⁂	4.10	<0.001⁂	0.47	0.50
**CV (variability) of tapping interval**										
SMT	0.07 ± 0.03	0.06 ± 0.02	0.10 ± 0.06	0.08 ± 0.02	6.07	0.02*	3.73	0.06	0.39	0.54
Maximum effort	0.12 ± 0.07	0.09 ± 0.03	0.15 ± 0.11	0.13 ± 0.09	2.80	0.10	1.51	0.22	<0.001	0.97
1.0 Hz	0.08 ± 0.04	0.07 ± 0.03	0.11 ± 0.08	0.09 ± 0.04	3.04	0.09	0.92	0.34	<0.001	0.36
2.0 Hz	0.07 ± 0.04	0.05 ± 0.02	0.16 ± 0.11	0.11 ± 0.09	7.69	<0.001⁂	0.29	0.59	0.02	0.88

Data are presented as mean ± standard deviation, Repeated measures two-way ANOVA was performed. *p < 0.05, ⁂*p* < 0.001.

The r-Amplitudes in both dominant and non-dominant hands were not significantly different between groups. In controls, r-Amplitude was 131.44 ± 13.42 mm (non-dominant hand)/123.91 ± 13.79 mm (dominant hand). In iNPH patients, the r-Amplitude was 137.20 ± 33.43 mm (non-dominant hand)/127.74 ± 20.63 mm (dominant hand). In frequency of taps, only the SMT condition showed significant difference between groups and maximum effort condition showed no significant difference by detected in the ANOVA. CVs of cl-Velocity in the dominant and non-dominant hands were significantly different for both controls and patients with iNPH, with the exception of the 1.0 Hz condition. There was no interaction effect (group × the dominant hand) observed in any parameter. There were significant differences between groups on almost all parameters of F-T. Finger tapping movements of the iNPH group were detected as being significantly impaired in amplitude and speed compared to the control group.

### The change of F-T performance by shunt surgery

**FIGURE 2:**
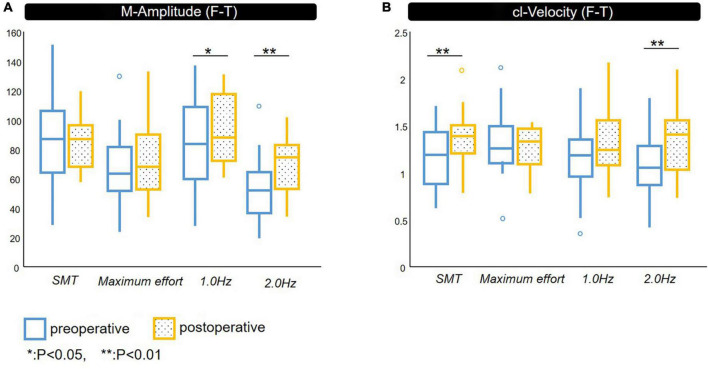
Spatiotemporal parameters of F-T. Comparison between pre- and post-shunt surgery with definite iNPH. **(A)** M-Amplitude of finger tapping, **(B)** cl-Velocity of finger tapping. preoperative, postoperative. iNPH, idiopathic normal pressure hydrocephalus; M-Amplitude, the mean of maximum amplitude; cl-Velocity, the line represents median values; *p < 0.05, **p < 0.01.

The comparison of paced F-T performance before and after shunt surgery in patients with iNPH is shown in Figure 2.

The M-Amplitude of F-T under 1.0 and 2.0 Hz conditions was significantly improved after shunt surgery.

The cl-Velocity of F-T showed significant improvement under SMT and 2.0 Hz conditions.

We did not identify significant improvement in CV after shunt surgery except for that of cl-Velocity of the non-dominant hand under 2.0 Hz condition.

### ROC analysis

For discrimination between iNPH and normal controls, the optimal cut-off levels of the F-T parameters under 2.0 Hz auditory stimuli conditions were determined using ROC analyses (Table 3). The optical cut-off of M-Amplitude was 56.4 mm, with 73% sensitivity and 94% specificity in the non-dominant hand and 57.1 mm, with 60% sensitivity and 100% specificity in the dominant hand, respectively.

**TABLE 3:** Areas under curves of m-amplitude and cl-velocity.

Measurement items	Dominant or non	AUC	95%CI	Optimal cut-off	Sensitivity (%)	Specificity (%)
M-amplitude (mm)	Non-dominant hand	0.83	0.60–0.93	56.4	73	94
	Dominant hand	0.81	0.60–0.92	57.1	60	100
cl-Velocity (m/s)	Non-dominant hand	0.84	0.62–0.95	1.20	73	38
	Dominant hand	0.88	0.70–0.96	1.44	87	75

The cut-off score obtained in cl-Velocity was 1.20 m/s, with 73% sensitivity and 38% specificity in the non-dominant hand and 1.44 m/s, with 87% sensitivity and 75% specificity in the dominant hand, respectively. The M-Amplitude of the optimal cut-off value was 41.1% (non-dominant hand)/44.7% (dominant hand) in patients with iNPH, as compared to r-Amplitude.

### Correlations between the parameters of F-T test and conventional assessment in patients with iNPH

**FIGURE 3:**
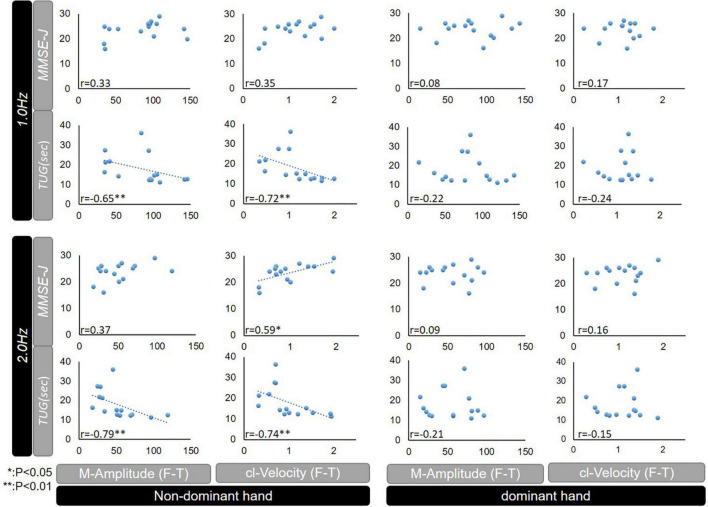
Correlations between MMSE-J or TUG, and F-T parameters (M-Amplitude or cl-Velocity) among patients with iNPH. MMSE-J, Mini Mental State Examination-Japanese; TUG, Timed up and go test; F-T, finger tapping; M-Amplitude, the mean of maximum amplitude; cl-Velocity, the maximum of closing movement velocity; iNPH, idiopathic normal pressure hydrocephalus; CV, coefficient of variation; NS, not significant; *p < 0.05, **p < 0.01.

The correlations between F-T parameters and MMSE-J and TUG under 1.0 and 2.0 Hz conditions are shown in Figure 3. Under the 1.0 Hz condition, both of the M-Amplitude and cl-Velocity correlated with TUG (Pearson’s correlation coefficient −0.65; *P* < 0.01 and −0.72; *P* < 0.01) in the non-dominant hand.

Under the 2.0 Hz condition, both of the M-Amplitude and cl-Velocity correlated with TUG (Pearson’s correlation coefficient −0.79; *P* < 0.01 and −0.74; *P* < 0.01), and cl-Velocity was further correlated with MMSE-J (Pearson’s correlation coefficient 0.59; *P* < 0.05) in the non-dominant hand.

## Discussion

The goal of this study was to investigate the optimal conditions for quantitative evaluation of repetitive F-T to help assess the function of iNPH patients. We evaluated repetitive simple F-T quantitatively in a control group and patients with iNPH. There are two main findings. First, the present study revealed that repetitive simple F-T was impaired in patients with iNPH compared to controls under the both not timing-regulated and timing-regulated conditions. Second, only the timing-regulated 2.0 Hz condition, post-operative improvement of the F-T test for iNPH was detected in this study for both M-Amplitude and cl-Velocity.

Regarding previous reports evaluating hand dexterity in iNPH, various methods including finger tapping have been reported. Lenfeldt et al. (2008) assessed manual dexterity by counting the total number of taps using sequential movement of four fingers (from the index to the little finger or reversed) and confirmed the improvement in the right hand after cerebrospinal fluid (CSF) drainage. Liouta et al. (2017) measured F-T speed according to the Halstead-Reitan neuropsychological test battery which uses a specially adapted manual tapper for the index finger and scored the number of taps (Reitan and Wolfson, 1993). They reported that the fine motor function of the right hand in iNPH improved after the lumbar puncture tap test. Behrens showed impairment of manual dexterity in iNPH using F-T, which improved after shunt surgery. The examiner assessed manual dexterity using four fingers on a small keyboard with digits to tap the digits in order and scored the correct taps (Behrens et al., 2014, 2019).

Based on these findings, all of these reports demonstrated the usefulness of the F-T test to assess motor function in iNPH. However, while these studies merely counted the number of finger taps, this study allowed us to measure F-T minutely and quantitatively by measuring the amplitude, velocity and other parameters in the present study.

Our rationale for choosing the 2.0 Hz rather than 1.0 Hz as the pacing frequency for timing-regulated of F-T is as follows. In behavioral and electrophysiological studies, the rate of about 2.0 Hz was shown to be a noteworthy frequency, and Mayville et al. (1999) provided evidence that a frequency of about 2.0 Hz is the band in which there is a shift from discrete to continuous control in repetitive finger movement with pacing stimuli (Toma et al., 2002). As Stegemöller et al. (2017) reported that in repetitive movements at frequencies slower than 2.0 Hz (e.g., 1.0 Hz), which are perceived as discrete movements, “control of discrete movements is considered to be preferentially mediated by cerebellar-premotor pathways,” while in repetitive movements at frequencies of about 2.0 Hz, which are perceived as continuous movements, “the control of continuous movement is mediated by the basal ganglia thalamocortical pathways.”

Based on these findings, the disturbance of repetitive movements at around 2.0 Hz, which are perceived as controlling continuous movements, reflects impairment from a dysfunction of the basal ganglia-thalamocortical network in PD (Ivry, 1996; Ivry and Spencer, 2004; Spencer and Ivry, 2005; Grahn and Brett, 2009; Stegemöller et al., 2017). Several reports have explained the implications of 2.0 Hz paced F-T in PD. Nowak et al. (2006) reported that fMRI findings suggest impairments of F-T in patients with PD reflect potential impairment in the supplementary motor area (SMA) (Nowak and Topka, 2006). Lenfeldt et al. (2008) suggested that the improvement of manual dexterity after CSF drainage in iNPH was related to the activation of SMA using fMRI. PD shares many characteristics with disturbances of iNPH mechanisms in the SMA related to frontal periventricular cortico-basal ganglia-thalamus-cortical pathways, which are connected with the impairment of finger movements with paced stimuli (Roland et al., 1980; Contreras-Vidal and Stelmach, 1995; Nowak and Topka, 2006; Witt et al., 2008; Genon et al., 2017; Liouta et al., 2017).

Previous studies have reported that the method of stimulus presentation is another important measurement condition. Witt et al. (2008) reported that all F-T tasks with pacing stimuli are commonly activated in the primary sensorimotor cortex, SMA, and anterior cerebellum by conjunction analysis, and especially, that the concordance within the dorsal premotor cortex (PMd) was lateralized based on task variation, with the auditorily-paced task in the right PMd. The right dorsal PMd is preferentially related to hand/finger movements and connected to both cognitive and motor regions in sequencing and rhythm-processing aspects common to finger movements, music, and language (Genon et al., 2017; Stegemöller et al., 2017). Therefore, using auditory paced F-T at the rate of 2.0 Hz to assess parkinsonism could be beneficial, because it is likely to provide high detectability of impairment.

In addition, the advantages of F-T may include the following. Paced F-T is easy for patients to perform in any position, sitting or lying down. Dawn et al. (2020) report that the timing-regulated condition is less susceptible to momentary affective states than the not timing-regulated condition, suggesting that it may be more appropriate to compare the performance of same subjects. According to Abe et al. (2017) hand dexterity may assess not only motor function but also cognitive function, and cross-sectional and longitudinal studies have shown that assessment of manual dexterity using pegs correlates with cognitive function in a healthy elderly. These reports suggest that the pacing F-T is a simple assessment, but that it may be used to assess not only motor function but also cognitive function.

There was another finding in this study, it was observed that CV of cl-Velocity in the repetitive F-T test differed between non-dominant and dominant hands. Previous imaging studies have reported that a hemispheric asymmetry in the functional activation of the motor cortex during contralateral and ipsilateral finger movements, especially in right-handed (Kim et al., 1993; Hayashi et al., 2008). Hayashi et al. (2008) reported that increase of contralateral activation and ipsilateral deactivation during the right-hand movement at high-frequency because the right-handed have the dominance of the left primary motor cortex. These studies suggest that the influence of non-dominant and dominant hand in right-handed should be taken into account in quantitative assessment of dexterous movements such as F-T test.

There are other barriers to the clinical application of this study. Quantitative evaluation of fingers is useful, but not as common as gait analysis. Previous studies reported quantitative evaluation of fingers using a 3D motion analyzer, which may be a potential alternative method (Horiba et al., 2019).

The limitation of our study is the small number of cases, further investigation is needed. Considering the possibility of applying it to tap tests and disease screening, the cut-off value obtained may be an indicator of the severity of impairment of hand dexterity in patients with NPH, which is an advantage of quantitative evaluation of F-T. Further studies with a larger number of subjects are needed to confirm the reproducibility of the repetitive 2.0 Hz F-T cut-off value and validate its usefulness as an indicator of motor impairment in iNPH. It may prove useful in revealing the features of upper limb parkinsonism in patients with iNPH in more detailed studies of finger tapping for differential clinical diagnosis.

## Conclusion

Quantitative assessment of F-T with 2.0 Hz auditory stimulation may be a useful and potentially supplemental screening method for motor assessment in patients with iNPH.

## Data availability statement

The raw data supporting the conclusions of this article will be made available by the authors, without undue reservation.

## Ethics statement

The studies involving human participants were reviewed and approved by Nagoya City University. The patients/participants provided their written informed consent to participate in this study.

## Author contributions

YS, MT, and MH conceived the idea of the study. YS, KS, and MH carried out the experiments. SK developed the statistical analysis plan and conducted statistical analyses. AK contributed to the interpretation of the results. TY and YN provided critical feedback and helped shape the research and analysis. YS drafted the original manuscript. MM, YU, and NM supervised the conduct of this study. All authors reviewed the manuscript draft and revised it critically on intellectual content and approved the final version of the manuscript to be published.
